# Editorial: Searching for Immune Tolerance Manipulating New Molecules and Exploiting New Concepts on Lymphocyte Biology

**DOI:** 10.3389/fimmu.2016.00027

**Published:** 2016-02-01

**Authors:** Diego Catalán, Karina Pino-Lagos

**Affiliations:** ^1^Programa Disciplinario de Inmunología, Facultad de Medicina, Instituto de Ciencias Biomédicas, Universidad de Chile e Instituto Milenio en Inmunología e Inmunoterapia, Santiago, Chile; ^2^Facultad de Medicina, Centro de Investigación Biomédica, Universidad de los Andes, Santiago, Chile

**Keywords:** immune tolerance, regulatory T cells, B cell tolerance, dendritic cells, transplantation, autoimmunity, costimulatory molecules, cytokines

**The Editorial on the Research Topic**

**Searching for Immune Tolerance Manipulating New Molecules and Exploiting New Concepts on Lymphocyte Biology**

This research topic was inspired in those still non-curable inflammatory conditions, such as autoimmune diseases and transplant rejection, based on the fact that immunologists worldwide are still searching for strategies to restore long-term immune tolerance. Thus, we gathered several researchers whom actively base their investigation lines in novel molecules and immune cell populations that can be exploited to design new strategies for the establishment or recovery of tolerance.

In the context of autoimmunity, intriguing is the role of interferon (IFN)-γ in the pathogenesis of multiple sclerosis (MS) and its animal model, the experimental autoimmune encephalomyelitis, which is reviewed in two articles contained in this edition (Ottum et al.; Arellano et al.). They focus on new evidence that help to explain the seemingly opposing effects of this cytokine over different central nervous system cells and on different stages of the disease, giving some important clues that can help to guide the potential therapeutic use of IFN-γ in MS patients. Other cytokine that recently has been focus of interest is IL-33, a molecule first described as an alarmin, but Gajardo-Carrasco et al. detail the plethora of now recognized functions in which IL-33 is involved, with special attention in T cell biology, adaptive immunity, tolerance, and immunological disorders.

Continuing with an update on molecules with pivotal immune function, Le Mercier et al. and Guo and Wang deliver us a solid snap shot on receptors and ligands with stimulatory and inhibitory immune activity, revising both classic and newest members, their contribution to disease and how they have been currently targeted to utilize them for therapeutic purposes. Special attention received the new Ig family member VISTA, which is presented as an interesting modulator of the immune response and with high potential for its exploitation in the clinic.

Similarly, the article by Iruretagoyena et al. addresses the immune regulatory aspects of vitamin D and its importance in controlling the development of autoimmune diseases. This review has a particular emphasis on the participation of this vitamin in the physiopathology of systemic lupus erythematosus (SLE) and gives an update on the latest data about vitamin D supplementation in SLE patients.

Regarding the use of immune cells with therapeutic purposes, this research topic contains five reviews that put the spotlight over the use of dendritic cells (DCs) and regulatory T cells (Tregs) as tools to treat immune-related conditions (including autoimmunity and transplant rejection). The article by Schinnerling et al. summarizes the recent advances in the description of intracellular pathways and transcriptional regulators that command the monocyte-derived tolerogenic human DCs differentiation program and propose candidate molecules that could be regarded as key in their tolerogenic functions. On the other hand, Maggi et al. examine one of the putative mechanisms of action of tolerogenic DCs; this is the induction of hyporesponsive or anergic CD4^+^ T cells. The authors review recent findings in the impact of CD4^+^ T cells anergy induction in animal models of autoimmune diseases development and progression, and discuss on the potential benefits of exploiting this mechanism for therapeutic purposes in humans. Similarly, Osorio et al. present a complete revision on DCs nature, from their origin, lineages, differentiation process, subtypes, and physiological role, linking these observations with diseases and mentioning current technological approaches to use them as a source for cellular therapy. On the other hand, Safinia et al. and Gregori et al. targeted human Tregs, describing extensively all their phenotypic characteristics, the different subpopulations identified to date based on certain surface markers and their mechanisms to drive immune suppression, and compiling simultaneously all the results from finished and ongoing clinical trials. In addition, both works discuss different aspects of human Tregs clinical grade manufacture and the variables that need to be improved to perfect the protocol, such as viability, antigen-specificity, cell expansion efficiency, and phenotypic/functional stability.

In parallel, the original article by Ruiz et al. proposes a modified protocol to favor mixed chimerism and further transplant acceptance in a preclinical model. Their novelty bases in the use of antigen-specific Tregs generated *in vitro* in the presence of IL-2, TGF-β, and retinoic acid (RA), in conjunction with previously established procedures as non-myeloablative irradiation and administration of immunosuppressant drugs. This group observed that the transfer of RA-Tregs facilitates donor-cells engraftment and allows for the acceptance of skin allografts, proposing the inclusion of Tregs as co-therapeutic tool. Another article reports how Soto et al. pinpoint at another cell population, frequently overlooked when it comes to tolerance mechanisms: B cells. Using systemic sclerosis (SSc) as a paradigmatic autoimmune disease with cellular and humoral components, the authors describe alterations in the expression levels of activator and inhibitor receptors on B cells from SSc patients that could contribute with the hyperactivated phenotype of these cells. They also demonstrate that IL-10-producing B cells and IL-10 secretion by stimulated B cells are reduced in SSc patients, which can imply that these patients have an impaired anti-inflammatory function on regulatory B cells, a subset specifically dedicated to promote tolerance to innocuous antigens. The restoration of the capacity of these cells to express adequate levels of protolerogenic molecules and regain their regulatory capability through novel or current B cell-targeted therapies could be a promising therapy for SSc or related autoimmune diseases.

Finally, the review by Parigi et al. brings us to a different face of immune tolerance, the one that keep us from mounting exacerbated immune responses against food antigens and commensal microbiota. Disruptions of the tolerogenic mechanisms displayed by a normal intestinal immune system can lead to severe conditions, such as food allergy or inflammatory bowel diseases. This review deals with the way how diet, breast milk, and solid food shape the immune system of newborns and defines the homeostasis in the intestinal microenvironment, thus conferring risk or protection for the future development of immune mediated diseases.

Overall, we achieved putting together a nice compilation on the current molecules and cell populations that are being aggressively targeted to restore immune tolerance in diseased patients. While many efforts are put in translational immunology, basic science immunologists continue working to satisfy these goals.

## Author Contributions

DC wrote the editorial for this research topic. KP-L coordinated this research topic and wrote the editorial.

## Conflict of Interest Statement

The authors declare that the research was conducted in the absence of any commercial or financial relationships that could be construed as a potential conflict of interest.

